# Assessment of the relationship between living alone and suicidal behaviors based on prospective studies: a systematic review and meta-analysis

**DOI:** 10.3389/fpubh.2024.1444820

**Published:** 2024-11-07

**Authors:** Zhipeng Luo, Jinfeng Wang, Xu Chen, Dejin Cheng, Yuanyuan Zhou

**Affiliations:** ^1^Department of Oncology, The Third People's Hospital of Yibin, Yibin, China; ^2^Yibin Vocational and Technical College, Yibin, China; ^3^West China Hospital, Sichuan University, Chengdu, China; ^4^The Second People's Hospital of Yibin, Yibin, China; ^5^Meishan Pharmaceutical College, Meishan, China

**Keywords:** living alone, suicide behaviors, prospective studies, systematic review, meta-analysis

## Abstract

Suicide, a global health concern, can be influenced by living arrangements. Hence, the objective of this systematic review was to assess the extent of the association between living alone and suicidal behaviors. We conducted a comprehensive search across eight databases for prospective studies. Hazard ratios (HR) and 95% confidence intervals were pooled using Stata software. Nine large-scale prospective studies with 3,663,205 participants proved eligible. The results of the meta-analysis showed that the pooled prevalence of living alone was 32%. Living alone was associated with suicide attempts and suicide death even after adjusting for all potential confounding factors (AHR = 1.45, 95% *CI*: 1.13–1.77; AHR = 1.27, 95% *CI*: 1.06–1.48). Compared to people who live with others, living alone increases the risk of suicide behaviors. Given the anticipated global rise in suicide rates and the growing prevalence of living alone, it is recommended to explore this issue on a broader scale.

**Systematic review registration:** PROSPERO, identifier: CRD42023464684, https://www.crd.york.ac.uk/prospero/display_record.php?ID=CRD42023464684.

## Introduction

Living alone represents a prevalent global social and psychological concern in contemporary society, constituting a significant element within the realm of negative social indicators. Over the past few decades, the prevalence of living alone has consistent global rise, this trend of living alone is particularly pronounced in developed nations. For instance, in European countries like Norway and Sweden, single-person households now constitute nearly half of all households (1). Between 1940 and 2020, there was a remarkable shift in the United States, with the overall proportion of individuals living alone surging from 7.7 to 27.6% (2). Furthermore, as per data from the 2021 China Statistical Yearbook, the year 2020 saw “one-person households” surpassing 125 million, accounting for more than a quarter of the total number of households (3).

Existing research indicate that there has been a growing interest in exploring the connection between living alone and individual health outcomes. Individuals who live alone are at an elevated risk of experiencing social isolation and loneliness, which can significantly undermine their psychological well-being, ultimately increasing the risk of premature mortality by roughly 30% (4).

Suicide is also a global health challenge, with over 800,000 people dying by suicide annually worldwide (5). In some countries, one in nine young individuals report having engaged in suicidal behaviors (6). Despite five decades of research, there has been limited progress in enhancing the prediction of suicidal behaviors. This underscores the urgent need for more targeted investigations into identifying specific risk factors associated with suicidal behavior (7). According to the interpersonal theory of suicide, individuals who live alone may experience failed belongingness due to unmet interpersonal needs and a lack of social connections, leading to psychological distress that heightens suicide risk (8). An increasing number of studies have noted that suicide risk varies with living arrangements, changes in family structure may contribute to the increase in suicide rates, and living alone may be an important objective risk factor for suicidal behaviors (9–12). A Danish follow-up study showed that living alone was a risk factor for subsequent suicide in men and women whose partners die (13). In addition, studies in Finland (14), the United Kingdom (15), and the Netherlands (16) have consistently reported that living alone is an independent risk factor for suicide.

While research has demonstrated that men are three times more likely to die by suicide than women, the specific nature of the relationship between gender, living alone, and suicidal behaviors remains unclear (17). Moreover, variations in social and cultural environments may influence living habits among individuals who live alone, potentially affecting the development of suicidal thoughts or behaviors (18). Research has shown that older adults individuals living alone who suffer from physical or mental illness are at a higher risk of suicide, particularly in the absence of social support and effective treatment (19, 20). Additionally, major negative life events, such as unemployment or financial difficulties, may serve as triggers for suicide among those living alone. A prospective study, a type of longitudinal research design, follows a cohort of similar individuals who differ in only one research factor over time, allowing researchers to examine the impact of that factor on outcomes such as death or disease progression. A longer follow-up period increases the number of observed outcome events, reveals long-term effects, and enables better control of bias and confounding variables, thereby enhancing the study’s accuracy and reliability.

### Current aims

This systematic review aimed to synthesize evidence on the relationship between living alone and suicidal behavior, and to quantify the strength of this association. The specific objectives of the review were to:

- Identify the prevalence of living alone based on prospective studies;
- Determine the extent of the association between living alone and suicidal behaviors based on prospective studies;
- Determine whether the relationship between living alone and suicidal behaviors varies with sociodemographic factors, follow-up years, or geographic location.

## Methods

The design and write of this review adhered to the Preferred Reporting Items for Systematic Reviews and Meta-Analyses (PRISMA) guidelines (21, 22). Additionally, the PROSPERO number is CRD42023464684.

### Search strategy

Major English and Chinese databases were systematically searched between January 1, 2000, and September 15, 2023, which include PubMed, Embase, Web of Science, Scopus, ProQuest, PsycINFO, CNKI, and WANFANG database. The search methodology used Boolean operators, combining ‘and’ with two core search terms: firstly, a set comprising ‘living alone’, ‘live alone’, ‘lives alone’, and ‘unaccompanied’ as alternatives; secondly, utilizing the wildcard ‘suicid*’ to capture a broad range of suicide-related concepts. A detailed retrieval strategy is provided in Supplementary material S1. The search was confined to prospective cohort studies published in either English or Chinese. To ensure the thoroughness of our literature search, we manually examined the reference lists of relevant systematic reviews or meta-analyses and also conducted an independent search on Google Scholar.

### Eligibility criteria

The inclusion criteria were as follows: (1) Population: individuals aged sixteen years and older, who do not live with others; (2) Exposure: living alone as a predictor of suicidal behaviors; (3) Outcome: reported measure of suicidal behaviors, include suicide attempt and suicide death; (4) Study design: prospective cohort studies.

The exclusion criteria were as follows: (1) Studies in languages other than English or Chinese; (2) Studies with were cross-sectional, case–control, qualitative, expert opinions, reviews, conference abstracts or those lacking available full texts, as well as duplicate studies; (3) Studies that offered incomplete data that could not be analyzed.

### Data extraction

Following the removal of duplicates via Endnote 20, we conducted a literature screening in Rayyan, a web application (23). The first and second authors independently assessed the eligibility articles based on the titles and abstracts. Subsequently, full text of the remaining studies will be scrutinized against all inclusion criteria, which required studies to provide information regarding the association between living alone and suicidal behaviors. To guarantee the reliability of the screening process, we mandate that the two authors must achieve an agreement rate of at least 95% during any evaluation stage. In instances where uncertainty arises, we immediately engage a third reviewer for discussion. If, after thorough deliberation, a consensus is still not reached, we escalate the matter to the corresponding author for a definitive resolution. This process ensures that every decision made throughout the evaluation is rigorous and well-founded. To ensure the accuracy and consistency of our data, we have adopted the following methods: Firstly, we clarify the extraction standards and rules prior to data extraction, ensuring a unified understanding among the two reviewers regarding the content to be extracted. Secondly, both reviewers independently extract data from the same segment, and their results are subsequently compared for consistency. Any discrepancies identified are discussed and resolved to reach a consensus. Lastly, we regularly arrange for a review session, where a third reviewer or expert verifies the extracted results. Finally, the following information was extracted independently by two reviewers: study characteristics (author, year of publication, country, cohort designation, follow-up duration); participant characteristics (age, sample size and sample type); outcome characteristics (the prevalence of living alone, the measure, forms and prevalence of suicidal behaviors). Furthermore, effect measures pertaining to the associations between living alone and suicidal behaviors were also be extracted. In cases of missing or unclear data, we initiated correspondence with the researchers, seeking further elaboration on both the methods and/or the results as dictated by the need for accuracy and completeness.

### Risk of bias assessment

Two independent reviewers assessed the methodological quality of the included studies, and any discrepancies resolved through consensus. The methodological risk of bias the included prospective studies was evaluated using the Newcastle-Ottawa Quality Assessment Scale (NOS) (24), which assigns scores ranging from 0 to 9 points, with scores of ≥7 points classified as indicative of high quality (25, 26). Meanwhile, we used Egger’s tests to identify publication bias.

### Statistical analysis

STATA software (version 17.0) was used for all statistical analyses, and a qualitative synthesis was conducted to summarize the characteristics of the included articles. If two or more adjusted effect size were provided in the same article, we would choose the most comprehensive adjusted effect size for pooling. Subgroup analyses were performed considering gender, continent, sample type and follow-up lengths. All ratios used in the subgroup analysis were adjusted for potentially confounding factors. The Cochrane *Q* test and *I*^2^ statistic were the two most often used indicator of heterogeneity. The model selected (fixed or random-effect) would depend on the magnitude of heterogeneity: A value of 0–25% indicates low heterogeneity and 26–50% indicates moderate heterogeneity, a fixed effects model was chosen; 51–100% indicates substantial heterogeneity, a random effects model was adopted (27, 28). Furthermore, the stability of the results was tested by leave-one-out sensitivity analyses, and a significance level of *p* < 0.05 (2-tailed) was taken as robustness after removing any observation.

## Results

### Search results

**Figure 1:**
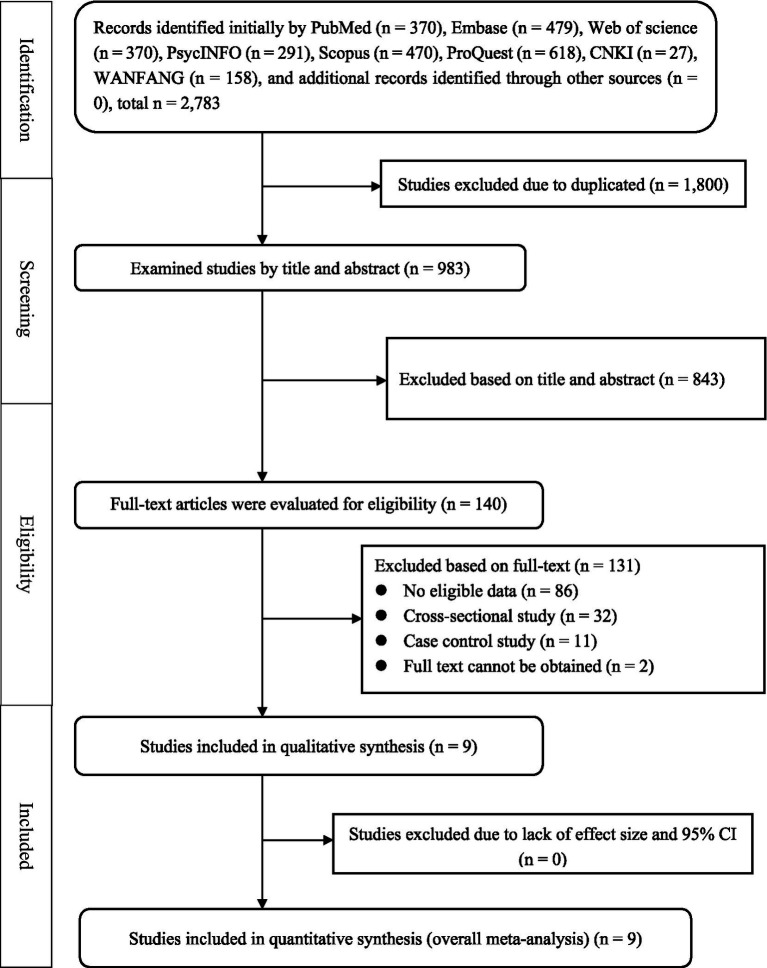
The process of literature search based on the PRISMA statement.

Figure 1 depicts the process of literature screening and selection using a PRISMA flow chart. Removal of the duplications left 983 records. After screening the title and abstract, 140 articles in the full-text evaluation stage, of which 9 studies were included in qualitative synthesis and meta-analyses.

### Studies characteristics

Table 1 provides an overview of the nine included studies. These studies were all prospective and were published between 2011 and 2021. The majority of them were conducted in European countries, with one study each from Canada (29) and Japan (18). The follow-up duration for these studies ranged from a minimum of 5 years to a maximum of 23 years, with an average duration of 11.2 years. The sample sizes varied considerably, ranging from 12,850 to 2,685,400 individuals, totaling 3,663,205 participants. Notably, except for one study that did not report specific gender counts (29), female participants outnumbered male participants in the remaining studies. In terms of the study populations, four studies focused on individuals diagnosed with various common mental disorders (14, 30–32), while another four investigated samples from the general population (15, 18, 29, 33). Only one study had a sample comprising stroke patients (34). Regarding living arrangements, the proportion of individuals living alone varied significantly, ranging from 4.51 to 55.55%. Data on suicidal behaviors were sourced from database registries or medical records in all the included studies, with suicide death consistently identified as the primary outcome measure. Additionally, three of the studies also simultaneously examined suicide attempts (31, 32, 34). The prevalence of suicide death in these studies ranged from 0.05 to 4.55%, while the prevalence of suicide attempt ranged from 0.45 to 5.8%.

**Table 1:** Characteristics of included studies.

Authors, year	Country	Design	Follow-up duration	Cohort designation	Sample (% Male)	Age/range Mean (SD)	Sample type	Living alone (%)	Suicide evaluation		
									Measure	Forms	Prevalence (%)
Aaltonen et al. (14)	Finland	Prospective study	24 years	Finnish population-based cohort	56,826 (44.32)	≥18	Patients with depressive disorder	29.40	Register data	SD	4.55
			1991–2014								
Burrows et al. (18)	Canada	Prospective study	10.6 years	Canadian census mortality follow-up study cohort	2,685,400	≥25	The general population	11.25	Census	SD	1.50
			1991–2001								
Eriksson et al. (34)	Sweden	Prospective study	12 years	The Swedish stroke register	220,336 (51.26)	≥18	Patients with stroke	47.49	Register data	SA	0.45
			2001–2012							SD	0.12
Hansson et al. (30)	Sweden	Prospective study	10 years	Swedish national quality register for bipolar affective disorder	12,850 (37.70)	47.8 ± 16.0	Patients with bipolar disorder	55.55	Register data	SD	0.70
			2004–2014								
Poudel-Tandukar et al. (18)	Japan	Prospective cohort study	13.2 years	The Japan public health center	104,528 (47.48)	40–69	The general population	4.51	Register data	SD	Male: 0.58
			1990–2005								Female: 0.21
Rahman et al. (31)	Sweden	Prospective cohort study	5 years	Nationwide register data	46,745 (33.68)	18–64	Patients with common mental disorders	41.90	Register data	SA	2.20
			2006–2010							SD	0.40
Schneider et al. (33)	Germany	Prospective cohort study	12 years	MONICA/KORA Augsburg surveys	12,888 (50.10)	25–74	The general population	21.31	Medical records	SD	0.30
Shaw et al. (15)	UK	Prospective cohort study	9 years	UK Biobank cohort	502,536 (46.50)	37–73	The general adult population	18.49	Register data	SD	0.05
			2006–2018								
Wang et al. (32)	Sweden	Prospective cohort study	5 years	Population-based prospective cohort	21,096 (40.05)	16–64	Patients with depressive disorders	53.40	Register data	SA	5.80
			2006–2010							SD	0.70

SD, Suicide death; SA, Suicide attempt; BDI, Beck Depression Inventory.

### Assessment quality of studies

The quality of included studies was assessed by the Newcastle Ottawa scale (NOS) for cohort studies. All the studies included in our analysis received quality assessment scores exceeding 7 points, classifying them as high-quality literature. However, four of these articles did not receive scores regarding the representativeness of their included samples due to their focus on specific disease populations (Supplementary Table S1).

### Results of meta-analysis

#### The prevalence of living alone

All included studies provided the proportion of people living alone. The results of the meta-analysis showed that the pooled prevalence of living alone was 32% (95% *CI*: 19–44%, *I*^2^ = 100%) (Table 2 and Supplementary Figure S1).

**Table 2:** Meta-analytic findings on the extent of the association between living alone and suicidal behaviors.

Estimate	K	N	Effect size	Pooled	95%*CI*	Q_within_	*I*^2^(%)	Tau	Tau^2^
Living alone	9	3,663,205	Prevalence	0.32	0.19–0.44	189882.63	100	0.2	0.04
Suicide attempt	3	288,177	Crude HR	1.58	1.18–1.98	8.20	82.66	0.30	0.09
	3	288,177	Adjusted HR	1.45	1.13–1.77	10.51	77.31	0.24	0.06
Suicide death	5	596,115	Crude HR	2.07	1.22–2.91	29.45	79.00	0.89	0.80
	8	3,442,869	Adjusted HR	1.27	1.06–1.48	22.15	59.48	0.22	0.05

K = Number of studies; N = Number of respondents; AHR: Adjusted Hazard Ratio; 95% CI = 95% Confidence Interval.

#### The association between living alone and suicidal behaviors

Table 2 presents the combined crude and adjusted Hazard Ratio (HR) estimates for the relationship between living alone and both suicide attempt and suicide death. This meta-analysis includes data from three studies, involving a total of 288,177 participants (31, 32, 34). The results reveal that, even after adjusting for all potential confounding factors, individuals who live alone exhibit a substantially higher risk of suicide attempt compared to those who do not live alone, the pooled adjusted HR (AHR) is 1.45 (95% *CI*: 1.13–1.77, *I*^2^ = 77.31%). Additionally, eight studies, involving a combined sample of 3,442,869 participants, investigated the association between living alone and suicide death (14, 15, 18, 29–33). Table 2 illustrates these findings, living alone is linked to the subsequent suicide death, with an AHR of 1.27 (95% *CI*: 1.06–1.48, *I*^2^ = 59.48%) (Supplementary Figure S2).

#### Subgroup analyses of the extent of the relationship between living alone and suicide death

Subgroup analyses were performed to assessment suicide death, considering factors such as gender (Female/Male), continent (Europe/America/Asia), sample type (The general population/Patients with psychiatric problems), and follow-up durations (≤10/>10 years). The consistent findings reveal that males who live alone face a significantly elevated risk of suicide death in comparison to those who do not, with an AHR of 1.26 (95% *CI*: 1.15–1.37, *I*^2^ = 59.56%). Significant difference was observed between females and males (*p* = 0.02). Furthermore, the study results did not exhibit significant variability across continents, sample types, and follow-up durations. Nonetheless, it’s noteworthy that individuals living alone in Europe exhibit a higher likelihood of suicide compared to those not living alone (AHR = 1.40, 95% *CI*: 1.01–1.80, *I*^2^ = 66.04%). In the general population, living alone was similarly associated with an increased risk of suicide compared to those not living alone (AHR = 1.33, 95% *CI*: 1.05–1.61, *I*^2^ = 47.14%). Furthermore, studies with follow-up periods exceeding 10 years indicated that individuals living alone are more likely to die by suicide than those not living alone (AHR = 1.20, 95% *CI*: 1.01–1.39, *I*^2^ = 51.20%). For further details, please refer to Table 3 and Supplementary Figures S3–S6.

**Table 3:** Subgroup analysis of the magnitude of the association between living alone and suicide death.

Subgroup	K	N	AHR	95% *CI*	Q_within_	*I*^2^(%)	*P* for difference
Gender							
Female	5	367,948	1.07	0.93–1.20	1.57	0	**0.02**
Male	5	308,792	1.26	1.15–1.37	9.89	59.56	
Continent							
Europe	6	652,941	1.40	1.01–1.80	13.80	66.04	0.80
America	1	2,685,400	1.23	0.88–1.58	–	–	
Asia	1	104,528	1.21	0.24–2.19	–	–	
Sample type							
The general population	4	3,305,352	1.33	1.05–1.61	11.26	47.14	0.71
Patients with psychiatric problems	4	137,517	1.23	0.80–1.67	6.98	64.30	
Length of follow-up (year)							
≤10	4	583,227	1.49	0.95–2.02	10.83	61.10	0.32
>10	4	2,859,642	1.20	1.01–1.39	10.43	51.20	

K = Number of studies; N = Number of respondents; AHR: Adjusted Hazard Ratio; 95% CI = 95% Confidence Interval. Bold values mean that the *P* value is < 0.05.

### Sensitivity analyses and publication bias

The results of the sensitivity analysis indicated that the estimated prevalence of living alone remained within the 95% confidence interval of the overall combined prevalence, regardless of the exclusion of any individual study. Furthermore, the sensitivity analysis revealed that the prevalence of living alone, upon the exclusion of each study, ranged from 0.28 (95% *CI*: 0.16–0.41) to 0.35 (95% *CI*: 0.23–0.47), with all *p*-values being 0.00 (Supplementary Figure S7). Similarly, after excluding each study, the hazard ratio between living alone and suicide death ranged from 1.21 (95% *CI*: 1.03–1.38) to 1.33 (95% *CI*: 1.05–1.61), and all *p*-values were 0.00 (Supplementary Figure S8). The results of Egger’s publication bias test for both the prevalence of living alone and the adjusted hazard ratio between living alone and suicide death were *p* = 0.053 and *p* = 0.177, respectively. Consequently, there was no clear evidence of publication bias (Supplementary Figures S9, S10).

## Discussion

Our study constitutes a systematic review of nine large-scale prospective studies conducted worldwide. The primary purpose was to examine whether living alone can predict subsequent suicidal behavior and to determine the strength of this association. This review emphasizes that, as the duration of living alone increases, individuals who live alone are more likely to engage in suicidal behavior compared to those who do not. Additionally, our meta-analysis reveals how factors such as gender, continent, sample type, and follow-up duration influence the extent of the association between living alone and suicide death.

### The global prevalence of living alone

A meta-analysis of nine large-scale prospective studies found that the prevalence of living alone was 32% (95% *CI*: 19–44%, *I*^2^ = 100%, *p* < 0.001), which is similar to previous findings, especially in the middle-aged and older adults population (35, 36). With declining birth rates and an aging global population, social arrangements have undergone significant changes. Many older adults individuals choose to live alone following the death of a spouse, particularly those without children or whose children live far away, leading to a marked increase in single-person households (37, 38). In some European countries, traditional family structures are shifting, with nuclear and single-person households becoming more common, while intergenerational cohabitation has become relatively rare. This cultural shift reflects society’s growing acceptance of independent and autonomous living (39, 40). Furthermore, values that emphasize individualism and self-fulfillment have heightened the demand for independent living, as people increasingly prefer to maintain a self-directed lifestyle. Economic improvements, particularly in developed countries, along with extensive social welfare systems, have fostered economic independence, contributing to a preference for living alone (41–43). Overall, social changes, growing economic independence, shifting cultural values, and other factors have all contributed to the increasing prevalence of living alone in modern society.

Although this study confirmed the prevalence of living alone, the high heterogeneity and narrow confidence intervals revealed by the *I*^2^ statistic have affected the credibility of our meta-analysis results. This significant heterogeneity may be attributed to the large sample sizes included in the analysis, which encompass a broad range of populations and span various age and gender distributions. Additionally, the diversity in living arrangements and sociocultural customs across different regions may also contribute to the observed heterogeneity. Given the presence of high heterogeneity, we employed a random effects model in our meta-analysis to better manage and account for the variability between studies, thereby enhancing the robustness and applicability of the analysis results.

### Living alone had long-lasting effects on suicidal behaviors

Previous studies have found that living alone increases the risk of suicidal behaviors, however, given the limitation that these analyses were based on cross-sectional studies, we will examine for the first time the relationship between living alone and the risk of suicidal behaviors in the context of longitudinal study data. The findings from this study suggest that, over time, living alone increases the risk of suicide attempts and suicide death by 45 and 27%, respectively. The association remained statistically significant even after controlling for a comprehensive set of covariates to the greatest extent possible, indicating that living alone maintains its independent influence.

The Interpersonal Theory of Suicide, developed by psychologist Thomas Joiner, provides a framework for understanding how living alone contributes to suicidal behavior (44). This theory emphasizes that suicidal thoughts arise from two interrelated but distinct factors: unmet belongingness and the perception of being a burden to others or society. It outlines the progression from ‘experiencing dangerous events’ to ‘developing suicidal thoughts’ and ultimately to ‘engaging in suicidal behavior’ (45). While individuals living alone may enjoy greater freedom and personal space, they are also at risk of experiencing loneliness, a key predictor of suicidal ideation. Prolonged loneliness can lead to a lack of belonging and feelings of hopelessness (46, 47). Additionally, those living alone may avoid social activities due to factors such as social anxiety or poor social skills, resulting in social isolation. This isolation not only exacerbates loneliness but also weakens social support networks, making it harder for individuals to seek help in times of need (48, 49). Furthermore, living alone may facilitate the means and opportunity for engaging in suicidal behavior.

However, there was significant heterogeneity in the association between living alone and suicide mortality. To address this, we conducted subgroup analyses to explore the sources of heterogeneity and discussed in detail the impact of potential confounders on the study results.

Subgroup analysis revealed that men living alone are more likely to die by suicide than women, consistent with previous research findings (50, 51). One valuable and important finding was that gender difference was statistically significant for the relationship between living alone and death by suicide. This disparity may be attributed to differences in social support, help-seeking behaviors, choice of suicide methods, and the impact of health and economic factors. Some studies have indicated the divergent preferences for suicide methods between men and women (52), men are more likely to choose highly lethal methods, such as jumping or hanging, while women are more inclined to use less fatal methods, such as drowning or medication overdose (14). Additionally, men living alone, especially older men, are more prone to undiagnosed or untreated physical and mental health issues, such as depression and alcohol dependence (53, 54), both of which are strongly associated with suicide risk.

Another subgroup analysis was related to geographic location. The majority of the included studies were from Europe, with only one study each from America and Asia. Therefore, caution is advised when interpreting the association between living alone and suicide mortality across different regions. Individuals living alone in Europe face a higher risk of suicide compared to those in America and Asia. This disparity may be attributed to a combination of sociocultural factors, including social isolation caused by individualistic culture, limited family support, the hidden of mental health issues, and reduced participation in social activities, particularly among older adults (55, 56). Many European countries are facing significant challenges related to population aging (57). As life expectancy increases, more and more older adults people choose to live alone or have to live alone. These older adults people often face problems such as widowhood, deteriorating physical health, and loss of social roles, which are recognized risk factors for suicide. In some European countries, suicide is historically or culturally viewed as a personal choice, and societal attitudes toward suicidal behavior are relatively tolerant (58). Similarly, in the Americas, many people who live alone place a high value on individualism and free will, which can influence their perspectives on suicide (59, 60). In some cases, suicide might be viewed as the ultimate expression of control over one’s own destiny. While this is not a mainstream belief, it reflects the cultural emphasis on personal freedom and autonomy. In Asia, particularly in Japanese culture, suicidal behavior is sometimes given a special meaning, especially under the influence of the “Bushido” spirit (61). This cultural concept may make it easier for some people to choose suicide as a solution when faced with difficulties or despair. Although modern society broadly promotes suicide prevention, this cultural legacy may subconsciously influence societal attitudes toward suicide, potentially resulting in inadequate prevention efforts or delayed social interventions.

Our subgroup results showed a puzzling finding that the association between living alone and suicide death was significant in the general population but not in those with psychiatric problems. This may be due to living alone has a stronger independent effect on suicide risk in the absence of other significant risk factors, whereas among people with mental problems, mental illness itself is the main driver of suicide risk. Therefore, the effect of living alone on suicide risk in these populations may be less prominent than the effect of mental illness. In other words, the presence of mental illness masks the effect of living alone. In the general population, living alone may be seen as a sign of isolation and helplessness, and psychological crises are not easily discovered by the outside world, increasing the risk of suicide. However, people with mental problems are usually considered a high-risk group, so society and family members may be more likely to be alert and intervene.

The results showed that variations in the length of follow-up did not exert a significant impact on the risk of suicide death among individuals living alone. The impact of follow-up time on suicidal behavior among individuals living alone may be obscured or weakened by various factors, including the individual’s living conditions, the effectiveness of intervention measures, and the methods of data collection and analysis. It’s crucial to emphasize that prolonged living alone does not necessarily lead to suicidal behaviors, and many individuals effectively manage the challenges associated with living alone while maintaining positive mental health (36).

### Strengths and limitations

In this study, we conducted a meta-analysis to assess the extent of the relationship between living alone and suicidal behaviors, aiming to draw a powerful conclusion. This meta-analysis providing comprehensive insights into the impact of living alone on the risk of suicidal behaviors. The study’s strengths include the following aspects: (1) We carried out a comprehensive search across eight major databases and included nine large-scale prospective studies, involving a cumulative total of 3,663,205 participants. The large population size enhances the generalizability of our findings; (2) All data included in the meta-analysis were sourced from database registries or medical records, enhancing the credibility of the study’s findings; (3) We conducted a range of subgroup analyses to identify potential sources of heterogeneity, and the pooled the crude and adjusted effect sizes separately, minimizing the probability of bias.

This study has some limitations. First, there is heterogeneity across cohort designs, such as sample size, duration of follow-up, etc., and the possibility of confounding by other unmeasured factors cannot be ruled out, so the interpretation and generalizability of the results will be limited. Secondly, lack of evidence to explore other subgroup effects that may be of interest, and considerable clinical heterogeneity in some subgroup analyses. For example, age and income should be considered as important factors affecting suicidal behaviors. However, subgroup analyses were not conducted due to inadequate original data. This might conceal some valuable information that affects the association between living alone and suicidal behavior. Furthermore, apart from considering the crude effect size, we chose to focus exclusively on the fully adjusted effect sizes for pooling, which may appear to overlook a substantial amount of information. Finally, the analysis was based on the dichotomous status of living alone or not, without discussion of the quality, commitment, or types of relationships people may be in, which may limit the generalizability of the findings.

## Conclusion

This study founded that compared to people who live with others, living alone increases the risk of suicide behaviors. The subgroup analysis revealed that gender, continent, sample type, and follow-up years were potential sources of heterogeneity, indicating that further research should focus on these factors. Additionally, future studies should thoroughly investigate the impact of age, economic status, and sociocultural factors on suicidal behavior among individuals living alone. Given that both living alone and suicide are significant social and psychological issues, future studies need to reveal the relationship between living alone and suicide in detail from the perspective of mixed-methods.

Public health departments should develop targeted policies and plans to improve social welfare systems. These should include enhancing the social support networks for individuals living alone to reduce loneliness and social isolation, strengthening education and publicity on suicide prevention to raise public awareness of the suicide risks of people living alone, and providing easily accessible psychological counseling and support services to assist individuals living alone in managing emotional distress and crises.

## Data Availability

The original contributions presented in the study are included in the article/Supplementary material, further inquiries can be directed to the corresponding author.
