# Supplementary material for: Assessment of the relationship between living alone and suicidal behaviors based on prospective studies: a systematic review and meta-analysis
Source: Front Public Health. 2024 Nov 7;12:1444820. doi: 10.3389/fpubh.2024.1444820 (PMC11580014; doi:10.3389/fpubh.2024.1444820)
Supplement: Supplementary file 1 [file Image_1.pdf]

Supplemental List

Supplementary 1	The search strategy and results	Page 2
Supplementary Table 1	Methodological quality of included cohort studies based on the Newcastle-Ottawa Scale	Page 6
Supplementary Figure 1	The prevalence of living alone	Page 7
Supplementary Figure 2	Pooled hazard ratio estimates of the association between living alone and suicidal behavior	Page 8
Supplementary Figure 3	Subgroup analysis by gender of the relationship between living alone and suicide death	Page 9
Supplementary Figure 4	Subgroup analysis by continent of the relationship between living alone and suicide death	Page 10
Supplementary Figure 5	Subgroup analysis by sample type of the relationship between living alone and suicide death	Page 11
Supplementary Figure 6	Subgroup analysis by follow-up length of the relationship between living alone and suicide death	Page 12
Supplementary Figure 7	Sensitivity analysis of the prevalence of living alone	Page 13
Supplementary Figure 8	Sensitivity analysis of the adjusted hazard ratio for living alone and suicide death	Page 14
Supplementary Figure 9	Egger's test for prevalence of living alone	Page 15
Supplementary Figure 10	Egger's test for publication bias based on the adjusted hazard ratio	Page 16

Supplement 1

Search strategy and results

Search strategy and results

Number of citations by each database register searched

**Databases**	**Citations**
PubMed	370
Web of Science	370
Embase	479
PsycINFO	291
Scopus	470
ProQuest	618
CNKI	27
WANFANG	158
Other sources	0
**Total (databases)**	**2783**

Full search strategy for each database

PubMed

#1 "living alone"[Title/Abstract] OR "live alone"[Title/Abstract] OR "lives alone"[Title/Abstract] OR "unaccompanied"[Title/Abstract]

#2 "Suicide"[Mesh] OR Suicid*[Title/Abstract]

#3 #1 OR #2

Web of science

#1 TS=("living alone" OR "live alone" OR "lives alone" OR unaccompanied)

#2 TS= suicid*

#3 #1 AND #2

Embase

#1 'living alone':ab,ti OR 'live alone':ab,ti OR 'lives alone':ab,ti OR unaccompanied:ab,ti

#2 'suicide'/exp OR suicid*:ab,ti

#3 #1 OR #2

PsycINFO

#1 TI ("living alone" OR "live alone" OR "lives alone" OR unaccompanied) OR AB ("living alone" OR "live alone" OR "lives alone" OR unaccompanied)

#2 MM "Suicide"

#3 TI suicid* OR AB suicid*

#4 #2 OR#3

#5 #1 AND #4

Scopus

#1 TITLE-ABS-KEY ("living alone" OR "live alone" OR "lives alone" OR unaccompanied)

#2 TITLE-ABS-KEY (suicid*)

#3 #1 AND #2

ProQuest

S1 title("living alone" OR "live alone" OR "lives alone" OR unaccompanied) OR abstract("living alone" OR "live alone" OR "lives alone" OR unaccompanied)

S2 title(suicid*) OR abstract(suicid*)

S3 S1 AND S2

China National Knowledge Infrastructure (CNKI)：

#1 主题=（独居）

#2 主题=（自杀）

#3 #1 AND #2

WANFANG：

#1 主题:(独居)

#2 主题:(自杀)

#3 #1 AND #2

Supplementary Table 1 Methodological quality of included cohort studies based on the Newcastle-Ottawa Scale

Study	Selection				Comparability of factors control	Outcome			Total score
	Representative of the exposed	Selection of the non-exposed	Ascertainment of exposure	Outcome of interest not presented at start		Outcome assessment	Long enough follow-up	Adequacy of follow-up	
Aaltonen *et al.* (2019)	-	*	*	*	**	*	*	*	8
Burrows *et al.* (2011)	*	*	*	*	**	*	*	*	9
Eriksson *et al.* (2015)	-	*	*	*	*	*	*	*	8
Hansson *et al.* (2018)	-	*	*	*	*	*	*	*	8
Poudel-Tandukar *et al.* (2011)	*	*	*	*	**	*	*	*	9
Rahman *et al.* (2014)	-	*	*	*	**	*	*	*	8
Schneider *et al.* (2014)	*	*	*	*	**	*	*	*	9
Shaw *et al* (2021)	*	*	*	*	**	*	*	*	9
Wang *et al.* (2015)	-	*	*	*	**	*	*	*	8

Note: *: one point awarded; **: two points awarded.

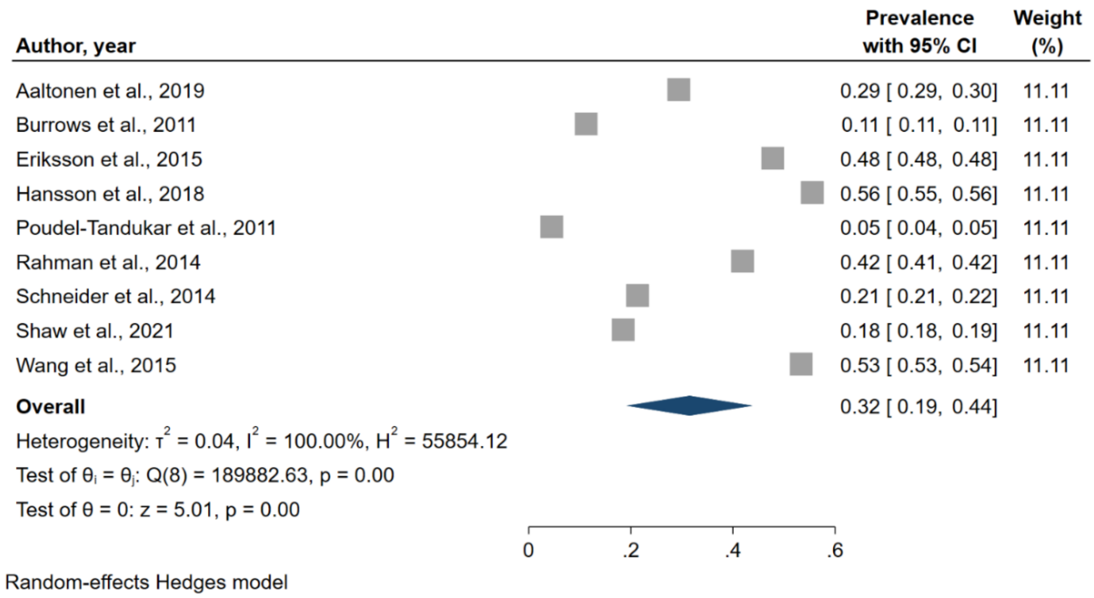

Supplementary Figure 1. The prevalence of living alone.

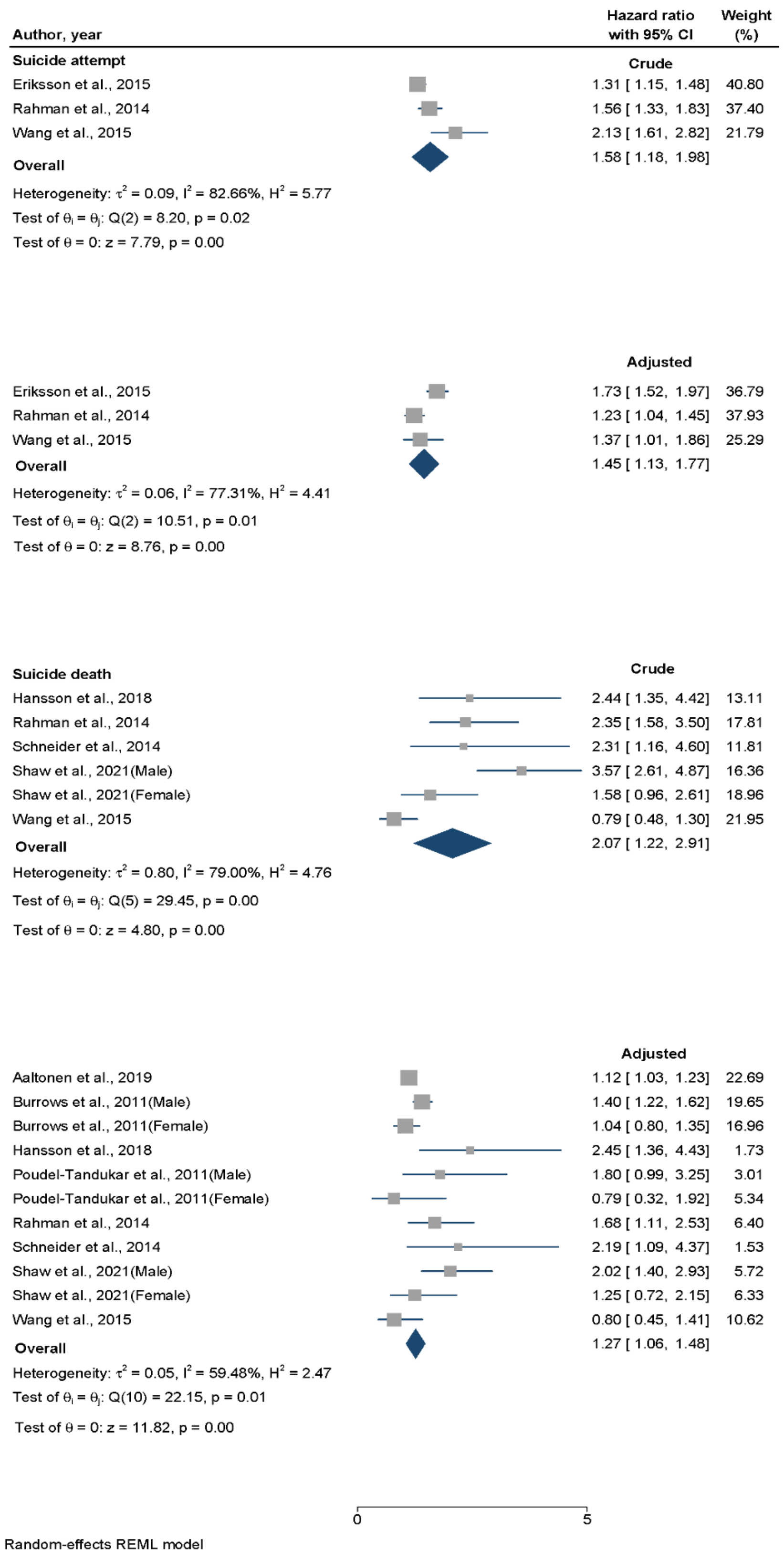

Supplementary Figure 2. Pooled hazard ratio estimates of the association between living alone and suicidal behaviors.

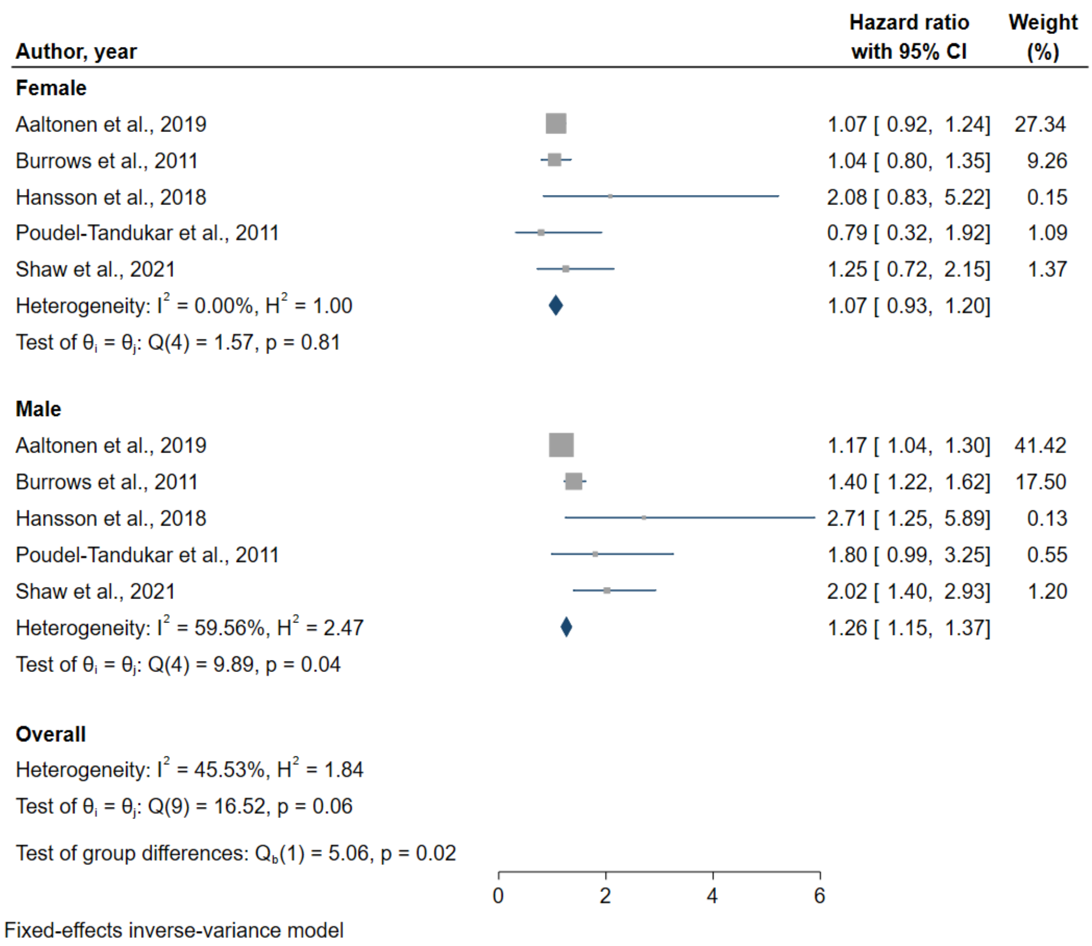

Supplementary Figure 3. Subgroup analysis by gender of the relationship between living alone and suicide death.

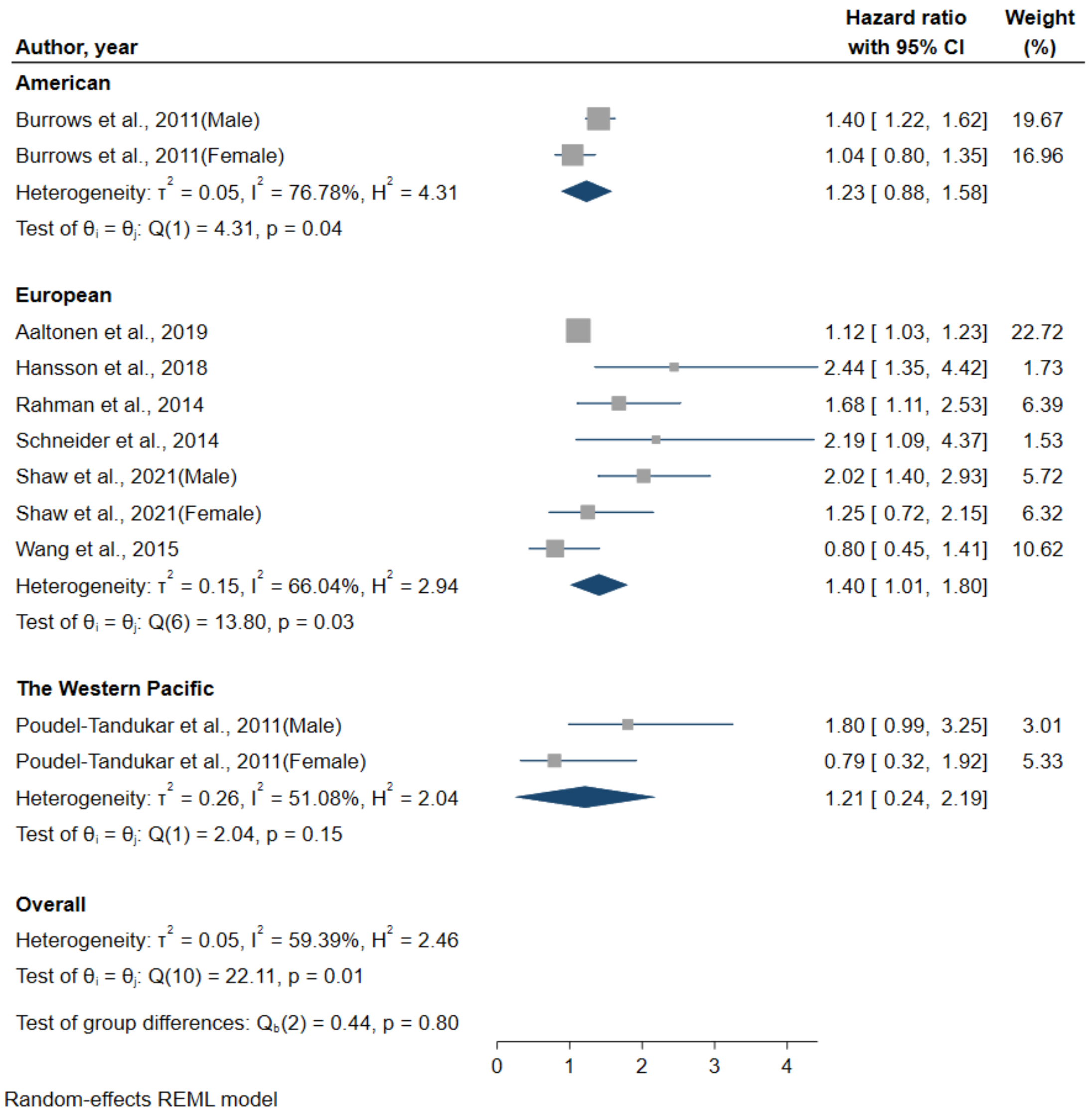

Supplementary Figure 4. Subgroup analysis by continent of the relationship between living alone and suicide death.

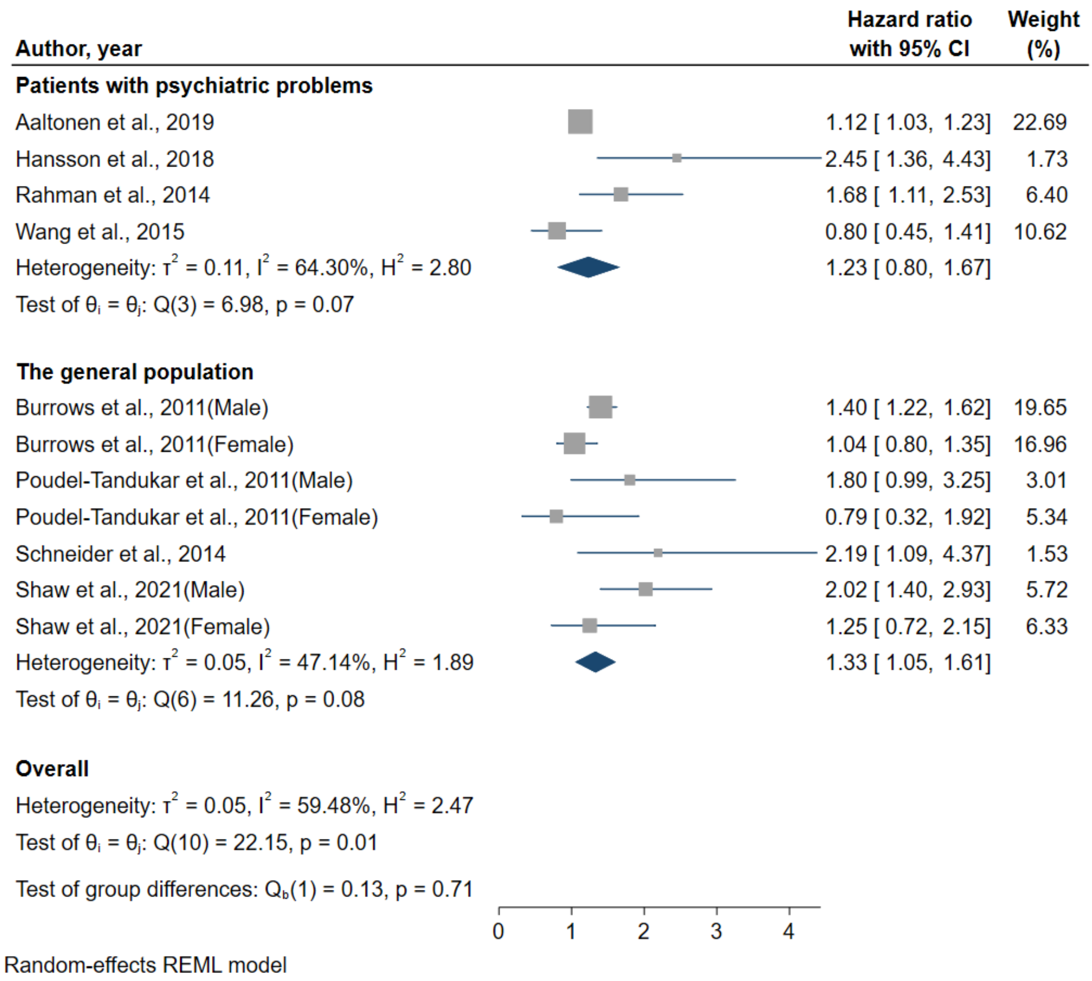

Supplementary Figure 5. Subgroup analysis by sample type of the relationship between living alone and suicide death.

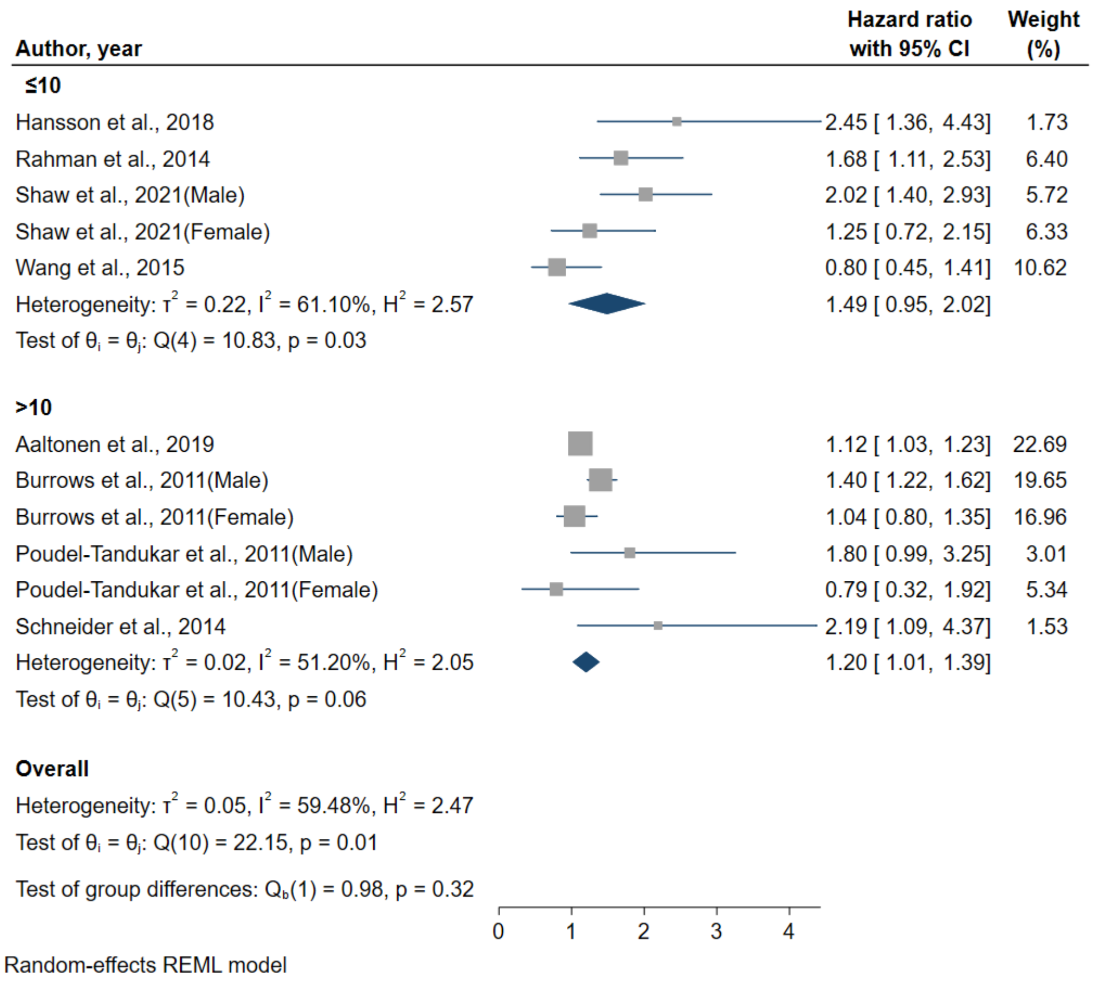

Supplementary Figure 6. Subgroup analysis by follow-up length of the relationship between living alone and suicide death.

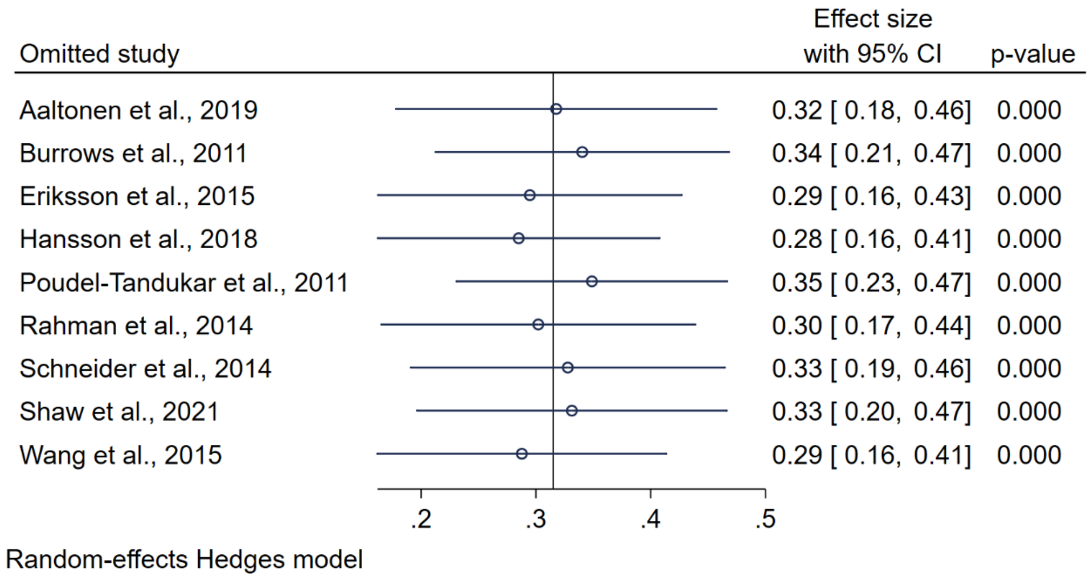

Supplementary Figure 7. Sensitivity analysis of the prevalence of living alone.

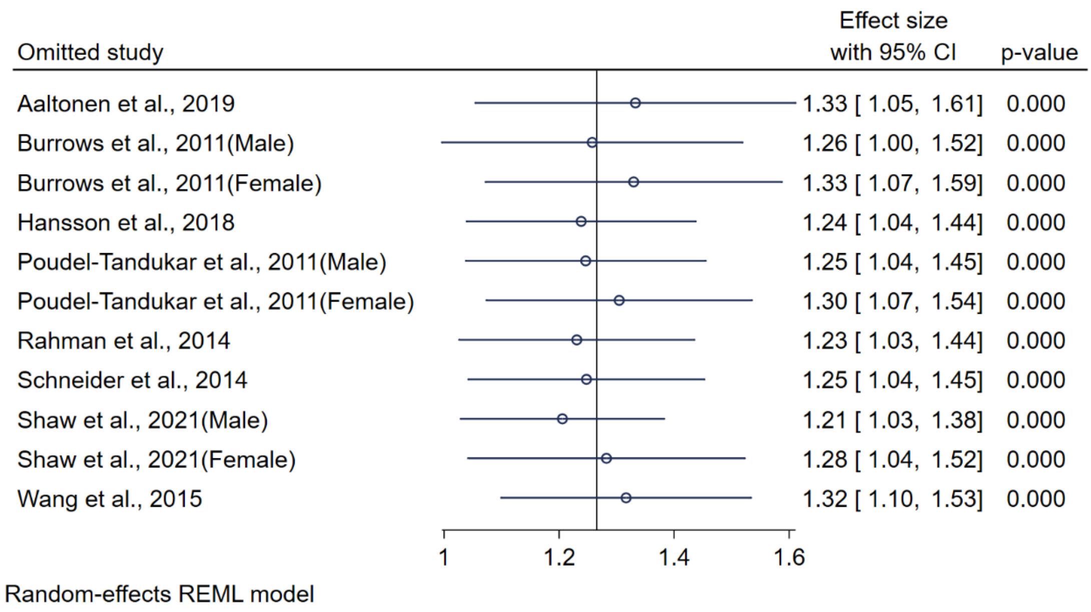

Supplementary Figure 8. Sensitivity analysis of the hazard ratio for living alone and suicide death.

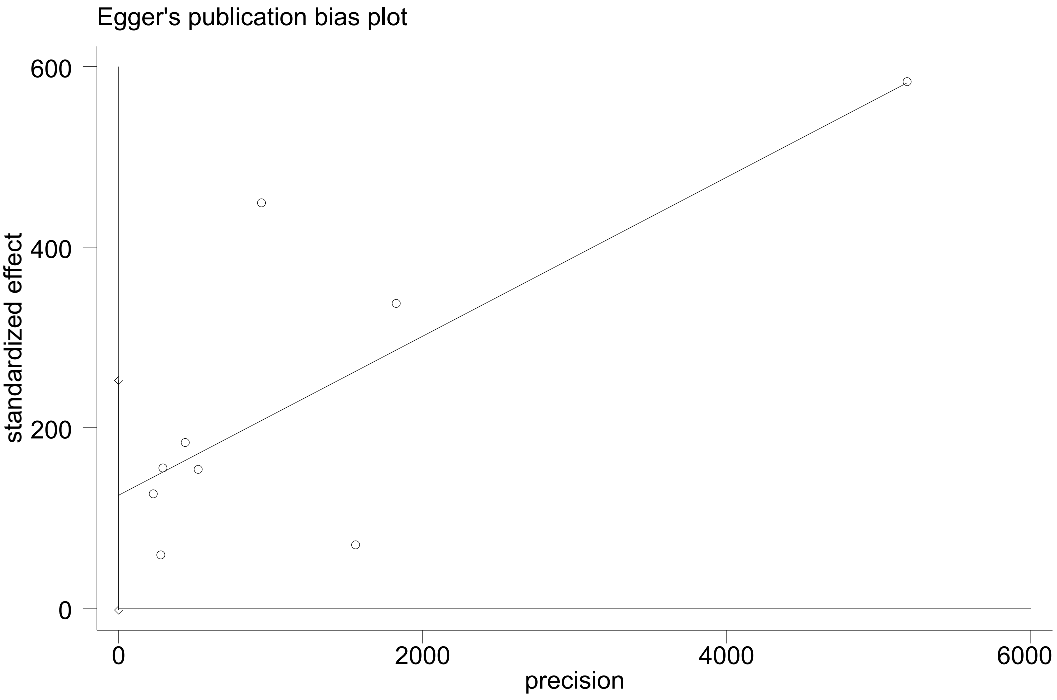

Egger's test

Std_Eff	Coefficient	Std. err.	t	P>|t|	[95% conf.	interval]
slope	.0880729	.0275676	3.19	0.015	.022886	.1532598
bias	125.1466	53.76328	2.33	0.053	-1.983409	252.2765

Supplementary Figure 9. Egger’s test for prevalence of living alone.

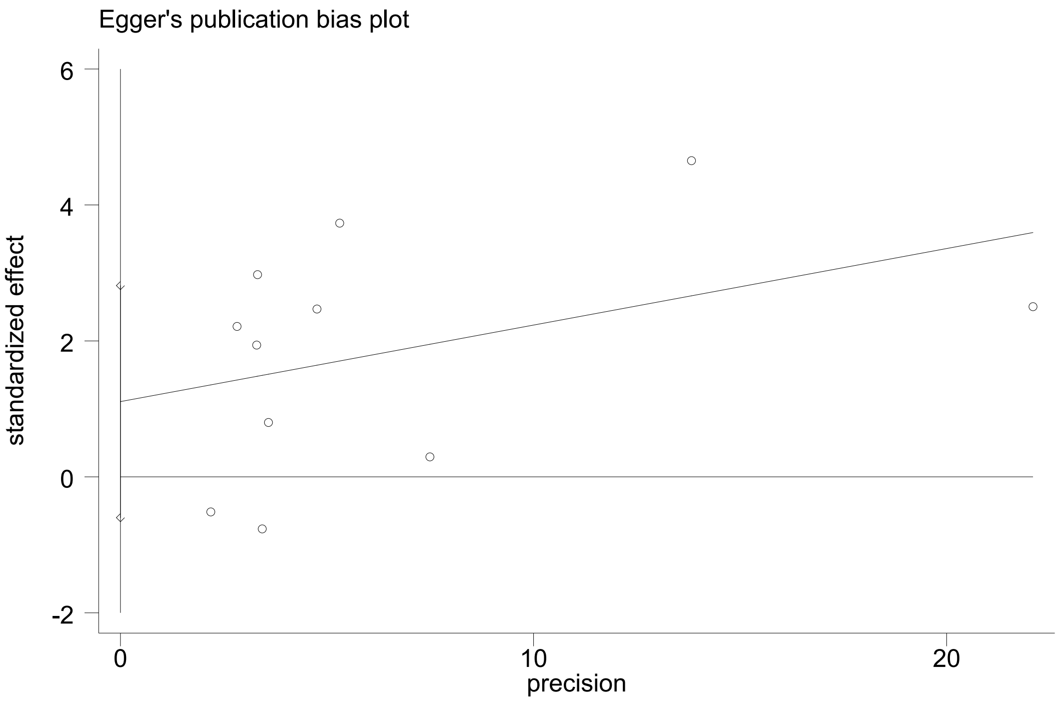

Egger's test

Std_Eff	Coefficient	Std. err.	t	P>|t|	[95% conf.	interval]
slope	.1125845	.0860872	1.31	0.223	-.0821583	.3073274
bias	1.106701	.7546392	1.47	0.177	-.6004116	2.813813

Supplementary Figure 10. Egger's test for publication bias based on the adjusted hazard ratio.
